# Dynamics of dendritic cells and T cells in HTLV-1-associated neuroinflammatory disease: implications in immunomodulatory therapies and diagnostic tools

**DOI:** 10.1186/1742-4690-8-S1-A187

**Published:** 2011-06-06

**Authors:** Sharrón L Manuel, George Makedonas, Michael R Betts, Jay Gardner, James J Goedert, Zafar K Khan, Pooja Jain

**Affiliations:** 1 Department of Microbiology and Immunology, and the Drexel Institute for Biotechnology and Virology Research, Drexel University College of Medicine, Doylestown, PA, 18902, USA; 2 Department of Microbiology and Immunology, University of Pennsylvania School of Medicine, Philadelphia, PA, 19104, USA; 3 Division of Cancer Epidemiology and Genetics, National Cancer Institute, Bethesda, Maryland, 20892-7335, USA

## 

HTLV-1-associated myelopathy/tropical spastic paraparesis (HAM/TSP), is a debilitating neurodegenerative disease characterized by a robust immune response including the oligoclonal expansion of cytotoxic T lymphocytes (CTLs) specific for the viral oncoprotein Tax. However, the underlying mechanism resulting in the disease process is currently unknown. The CTL response is affected by many factors including the efficiency of epitope processing and presentation. In this respect, dendritic cells (DCs), the most potent antigen presenting cells, have long been recognized as key regulators of the immune system. We have previously demonstrated that DCs are capable of priming a pronounced Tax-specific CTL response in naïve PBLs and in HLA-A2 transgenic mice. Since DCs are such crucial cells of the immune system, an extensive assessment of their function and interaction with T cells in HAM/TSP is critical. Therefore, utilizing a newly standardized DC and pre-standardized T cell polychromatic antibody cocktails, we have investigated the immune activation of these cells in HTLV-1 infected samples from the Jamaican region including the seronegative controls, asymptomatic carriers (ACs), and HAM/TSP patients. The extensive immune cell profiling was compared to the matched proviral loads and Tax mRNA levels leading to the identification of unique signatures distinguishing ACs from HAM/TSP patients. Collectively, these studies possess great potential to enable immune cell monitoring and development of diagnostic and therapeutic strategies for the HTLV-associated neuroinflammatory disease.

